# Human Dendritic Cell Maturation Is Modulated by *Leishmania mexicana* through Akt Signaling Pathway

**DOI:** 10.3390/tropicalmed9050118

**Published:** 2024-05-17

**Authors:** Jorge Rodríguez-González, Arturo A. Wilkins-Rodríguez, Laila Gutiérrez-Kobeh

**Affiliations:** 1 Laboratorio de Estudios Epidemiológicos, Clínicos, Diseños Experimentales e Investigación, Facultad de Ciencias Químicas, Universidad Autónoma Benito Juárez, Oaxaca C.P. 68120, Mexico; jorge.r.g@outlook.com; 2 Unidad de Investigación UNAM-INC, División de Investigación, Facultad de Medicina, Universidad Nacional Autónoma de México-Instituto Nacional de Cardiología “Ignacio Chávez”, Juan Badiano No. 1, Col. Sección XVI, Tlalpan, Mexico City C.P. 14080, Mexico; wilkins_aar@comunidad.unam.mx

**Keywords:** Akt, CD86, dendritic cells, ERK, IL-12, *Leishmania mexicana*, maturation, MHC-II

## Abstract

Dendritic cells (DC) along with macrophages are the main host cells of the intracellular parasite *Leishmania*. DC traverse a process of maturation, passing through an immature state with phagocytic ability to a mature one where they can modulate the immune response through the secretion of cytokines. Several studies have demonstrated that *Leishmania* inhibits DC maturation. Nevertheless, when cells are subjected to a second stimulus such as LPS/IFN-γ, they manage to mature. In the maturation process of DC, several signaling pathways have been implicated, importantly MAPK. On the other hand, Akt is a signaling pathway deeply involved in cell survival. Some *Leishmania* species have shown to activate MAPK and Akt in different cells. The aim of this work was to investigate the role of ERK and Akt in the maturation of monocyte-derived DC (moDC) infected with *L. mexicana*. moDC were infected with *L. mexicana* metacyclic promastigotes, and the phosphorylation of ERK and Akt, the expression of MHCII and CD86 and IL-12 transcript, and secretion were determined in the presence or absence of an Akt inhibitor. We showed that *L. mexicana* induces a sustained Akt and ERK phosphorylation, while the Akt inhibitor inhibits it. Moreover, the infection of moDC downregulates CD86 expression but not MHCII, and the Akt inhibitor reestablishes CD86 expression and 12p40 production. Thus, *L. mexicana* can modulate DC maturation though Akt signaling.

## 1. Introduction

Leishmaniasis is a tropical neglected disease distributed in tropical and subtropical regions, which is unfortunately linked to poverty. It is estimated that leishmaniasis affects nearly 14 million people around the world, with an estimated annual incidence of 700,000 to 1 million people [1]. Different species of the genus *Leishmania* are transmitted through the bite of a hematophagous female phlebotomine sand fly (*Lutzomyia* in America and *Phlebotomus* in the Euroasiatic and African continents) and cause a wide spectrum of clinical forms. These different clinical forms can be mainly grouped in three: cutaneous, mucocutaneous, and visceral leishmaniasis, the last one being the most severe [2]. The three clinical forms are present in Mexico, although the most prevalent form is cutaneous leishmaniasis caused by *Leishmania mexicana*. Interestingly, this *Leishmania* species is capable of causing two diametrically opposite forms of cutaneous leishmaniasis: localized (LCL) and diffuse (DCL) cutaneous leishmaniasis. This phenomenon has been associated with both parasite virulence and immunologic state of the patient together with its genetic background [3]. Other *Leishmania* species, such as *L. infantum* and *L. braziliensis,* can cause visceral and mucocutaneous leishmaniasis, respectively. *L. braziliensis* can also cause cutaneous leishmaniasis [2].

The *Leishmania* life cycle initiates when the parasites are transmitted through the bite of the sand fly that inoculates metacyclic promastigotes when feeding on a mammalian host. These are swiftly phagocytosed by neutrophils and resident macrophages. Inside the host cell, metacyclic promastigotes transform into amastigotes, which intensely divide and are finally released and phagocytosed by neighbor cells such as macrophages and DC. For the parasite to reproduce in the hostile microenvironment of macrophages and DC, it is imperative to inhibit or delay the microbicidal mechanisms of host cells, such as phagosome–lysosome fusion, reactive oxygen (ROS) and nitrogen (RNS) species synthesis, and IL-12 and IFN-γ secretion [3,4,5]. DC, along with macrophages, are the main *Leishmania* host cells; additionally, DC are responsible for polarizing the immune response to a Th1 profile through the secretion of IL-12. DC are the most potent antigen-presenting cells (APC) due, among other things, to their capacity to prime naïve T cells [6,7]. DC transit though two functional phases, “immature” and “mature”, which are characterized by several features, but the ability to phagocytose and to activate antigen-specific naïve T cells are the hallmarks of immature and mature DC, respectively [8,9,10]. The process of DC maturation is complex and triggered by several factors, including perturbations in tissue homeostasis caused by detection of pathogen-associated molecular patterns (PAMPs) or danger-associated molecular patterns (DAMPs) [11,12]. Maturation allows DC to migrate from peripheral regions to T-cell areas in secondary lymphoid organs where antigen presentation occurs. Several metabolic, cellular, and gene transcription programs are activated to achieve the goal [13,14,15,16,17,18]. One signaling pathway that has been shown to participate in DC maturation is mitogen-activated protein kinases (MAPK) [19]. Among different signaling cascades, the MAPK pathway plays a central role in cell proliferation, differentiation, apoptosis, angiogenesis, and tumor metastasis [20,21,22,23]. Four MAPK cascades, each one consisting of at least three levels of phosphorylation, namely MAP3K, MAPKK, and MAPK, have been identified in eukaryotic cells [24]. These are ERK, c-Jun N-terminal kinases JNK/stress-activated protein kinase (SAPK), p38 MAPK, and ERK5. Particularly, ERK/MAPK is the most thoroughly studied MAPK signaling pathway, playing a pivotal role in cell proliferation and differentiation and functioning as a central hub in the cell signal transduction network [25,26,27,28]. During DC maturation, ERK, JNK, and p38 are phosphorylated under several stimuli and differentially regulate all aspects of the phenotypic maturation, cytokine production, and functional maturation of monocyte-derived dendritic cells (moDC) [19]. Another signaling pathway deeply involved in cell survival and proliferation is the phosphatidylinositol-3 kinase/protein kinase B/Akt (PI3K/Akt). Akt is a serine/threonine kinase expressed as three isozymes: Akt1, Akt2, and Akt3 in mammalian cells. It is activated following the activation of the (PI3K)-coupled growth factor receptors, phosphorylates multiple substrates, and intervenes in crucial functions such as cell survival, proliferation, DNA repair, and anabolic processes [29,30,31,32]. MAPK and Akt are pathways that transit in parallel, but different cross-talk points have been demonstrated [33]. 

Moreover, the preponderant role of DC maturation in the infection with *Leishmania* has been demonstrated in the murine model of infection of susceptible BALB/c and resistant C57BL/6 mice with *L*. *major.* The model has shown that DC play an essential role in the activation of naïve T cells thanks to their maturation process. It is characterized by an increase in the costimulatory molecules CD40, CD80, and CD86, along with IL-12 production that potentiates the activation of naive T lymphocytes. This results in a Th1 response with an elevated IFN-γ production and species-specific cytotoxic T lymphocytes (Tc1), which finally leads to the activation of macrophage microbicidal mechanisms to achieve the elimination of the parasite [5,34]. Macrophages infected with *Leishmania* do not activate microbicidal mechanisms such as nitric oxide (NO) and ROS production, and at the same time, IL-12 secretion is blocked even in the presence of stimuli like lipopolysaccharide (LPS) or IFN-γ [35,36]. Nevertheless, the infection with *L*. *mexicana* does not lead to the same outcome. It has been shown that the infection of murine macrophages or DC with *L*. *mexicana* inhibits IL-12, while in DC, it delays maturation by inhibiting MHCII, CD86, and CD54 expression [37]. Previously, it was shown that the infection of CBA mice bone marrow-derived DC with *L. mexicana* amastigotes and promastigotes inhibits DC maturation. There is no increase in surface markers such as CD54, CD86, and MHC-II, and the production of IL-12 is inhibited. Interestingly, the infection of DC does not eliminate its maturation capacity, given that they manage to mature if stimulated with LPS/IFN-γ [38]. It has been observed that the infection with *L*. *mexicana* promastigotes of immortalized DC derived from resistant C57BL/6 mice also inhibited DC maturation. This has been associated with a rapid activation of tyrosine phosphatases that could be directly related to MAPK, along with the inhibition of transcription factors such as AP-1 and NF-κB [39]. However, there is no more extensive information about the effect of the infection of human-derived DC with *L. mexicana* in the process of maturation. Our group has demonstrated that the infection of moDC with *L*. *mexicana* amastigotes and promastigotes inhibits the activation of p38 and JNK MAPK and, on one hand, activates PI3K/Akt as strategies to inhibit moDC apoptosis induced with camptothecin [40,41]. On the other hand, it has been shown that the infection of murine macrophages with *L*. *amazonensis* induces a sustained activation of Akt, and the pharmacological inhibition of this pathway restores IL-12 production [42]. Still, the studies with human DC infected with *L*. *mexicana*, the causal agent of leishmaniasis in Mexico, are limited. In this work, we aimed to determine the participation of Akt signaling pathway in the maturation of moDC during infection with *L. mexicana* metacyclic promastigotes. 

Our results show that the infection of moDC with *L. mexicana* metacyclic promastigotes induces a sustained Akt and ERK phosphorylation, while an Akt inhibitor inhibits it. Moreover, the infection of moDC downregulates some features of DC maturation such as IL-12 p40 transcription and secretion and CD86 expression but not MHCII presence. Interestingly, the inhibition of Akt reestablishes CD86 expression and IL-12 production. Thus, *L. mexicana* can modulate DC maturation through Akt signaling.

## 2. Materials and Methods

### 2.1. Culture of Leishmania mexicana

*L. mexicana* Lac strain (MHOM/MX/2011/LAC) was used throughout the experiments and was chosen because of its importance as the causal agent of cutaneous leishmaniasis in Mexico. The strain was isolated from a patient diagnosed with LCL after returning from a trip to the Lacandona Rainforest region of the Mexican state of Chiapas. It was confirmed to be *L. mexicana* via restriction fragment length polymorphism analysis of polymerase chain reaction amplicons of the internal transcribed spacer 1 (ITS1) and of the 5.8S ribosomal RNA, as previously described [43]. All the infections were carried out with promastigotes enriched in the metacyclic phase. This process was performed according to Bates and Tetley [44]. In essence, 1 × 10^5^ metacyclic promastigotes/mL were differentiated from amastigote axenic cultures in medium 199 (Sigma-Aldrich; Merck KGaA, Darmstadt, Germany) supplemented with 10% heat-inactivated fetal bovine serum (FBS) (Biowest), 100 U/mL penicillin, 100 μg/mL streptomycin, and 2 mM _L_-glutamine (Gibco; Thermo Fisher Scientific, Inc., Waltham, MA, USA) at pH 7.2. Subcultures were performed on day 3 of culture at a density of 1 × 10^5^ parasites/mL of supplemented Grace’s medium (Gibco; Thermo Fisher Scientific, Inc. Waltham, MA, USA) with 10% FBS, 100 U/mL penicillin, 100 µg/mL streptomycin, and 2 mM _L_-glutamine at pH 5.4 to encourage metacyclogenesis [45]. Finally, on the fifth day of culture, metacyclic promastigotes were harvested by centrifugation at 2000× *g* for 10 min, washed three times with Dulbecco’s–phosphate-buffered saline (D-PBS) (Biowest, Riverside, MO, USA), and counted using a hemocytometer after fixing in 0.2% glutaraldehyde solution in D-PBS.

In order to maintain the virulence of the *L. mexicana* Lac isolate, susceptible Balb/c mice were periodically infected with amastigotes. For this purpose, 1 × 10^7^ axenically cultured amastigotes were washed with D-PBS and were inoculated in the footpad. Once the footpad lesion developed, the mouse was sacrificed, and the infected limb was excised. The infected foot was macerated through a 100 mm nylon mesh (BD Falcon, Bedford, MA, USA) using a syringe plunger and then centrifuged at 100× *g* for 5 min at 20 °C. The supernatant was recovered, centrifuged at 2000× *g* for 10 min at 20 °C, the pellet was washed 3× with D-PBS, and the isolated amastigotes were seeded at a density of 1 × 10^5^ parasites/mL of supplemented Grace’s medium at pH 5.4 and cultured at 32 °C [46]. Amastigotes that were cultured for 7 days were used to infect more mice. 

### 2.2. Monocyte-Derived Dendritic Cells (moDC) Culture

Monocyte-derived dendritic cells (moDC) were differentiated from CD14^+^ monocytes as previously described [47,48]. Peripheral blood monocytes were obtained from buffy coats from healthy donors, which were kindly supplied by the blood bank of the National Institute of Cardiology “Ignacio Chávez”. Informed consent forms were signed for the use of blood samples according to the Declaration of Helsinki, and the local scientific and ethics committees approved the protocol. Buffy coats were diluted 1:2 con D-PBS to form a gradient with Histopaque-1077 (Sigma-Aldrich; Merck KGaA, Darmstadt, Germany). The gradient was centrifuged at 400× *g* at 24 °C for 45 min. Afterwards, the mononuclear cells layer was recovered, and cells were washed three times with D-PBS at 300× *g* at 4 °C for 10 min. Then, mononuclear cells were incubated with blocking buffer (D-PBS, 2 mM EDTA, and 0.5% BSA cell culture grade) at 4 °C for 15 min. Immediately afterwards, anti-CD14 magnetic microbeads (Miltenyi Biotec, Bergisch Gladbach, Germany) were added and incubated for 20 min at 4 °C. Mononuclear cells incubated with the microbeads were washed one more time. Anti-CD14-marked cells were separated through a LS magnetic column coupled to a magnet (Miltenyi Biotec, Bergisch Gladbach, Germany). The column was washed three times with 3 mL of D-PBS to eliminate CD14^−^ cells and then separated from the magnet, and CD14^+^ monocytes were recovered with the help of a plunger and D-PBS. Afterwards, monocytes were counted and seeded at a density of 1 × 10^6^ cells/mL on 24-well tissue culture-treated plates in RPMI-1640 medium with stable glutamine (Biowest, Riverside, MO, USA) supplemented with 10% FBS, 100 U/mL penicillin, 100 µg/mL streptomycin, and 50 µM 2-mecaptoethanol at pH 7.2 (R-10 medium). The following day, 50% of the medium of each well was replaced with fresh R-10 medium enriched with 500 U/mL granulocyte macrophage–colony-stimulating factor (GM-CSF) and 1000 U/mL IL-4 (BD Biosciences, Franklin Lakes, NJ, USA) to encourage the differentiation of CD14^+^ monocytes to immature moDC. During days 2 and 4 of culture, 50% of the medium was replaced with fresh R-10 enriched with cytokines. To obtain an immature phenotype, moDC were harvested on days 5–6 of culture and used for all the assays. 

### 2.3. moDC Infection and Treatment 

Then, 1 × 10^6^ moDC obtained from days 5–6 of culture were seeded in 24-well tissue culture plates. Re-seeded cells were left to rest for at least 4 h in a humid atmosphere at 37 °C and 5% CO_2_/95% air. Promptly, moDC were infected with *L. mexicana* metacyclic promastigotes at a parasite/cell ratio of 10:1 and incubated for 2 h at 26 °C. Once the incubation time was over, the plate was transferred again to a humid atmosphere at 37 °C and 5% CO_2_/95% air for 24 h. 

To evaluate the participation of the kinase Akt in the process of maturation of infected moDC, the inhibitor VIII specific for Akt (Sigma-Aldrich; Merck KGaA, Darmstadt, Germany) was added to moDC at a 10 µM concentration and incubated in a humid atmosphere at 37 °C with 5% CO_2_/95% air for 2 h. For some experiments, the ERK inhibitor FR180204 (sc-203945) (Santa Cruz Biotechnologies, Santa Cruz, CA, USA) was also used at a 10 µM concentration. Both inhibitors were dissolved in DMSO. Afterwards, *L*. *mexicana* metacyclic promastigotes were added and incubated at 26 °C for 2 h. Once the incubation time was over, the plate was transferred to a humid atmosphere at 37 °C and 5% CO_2_/95% air for 18 h. 

### 2.4. moDC Infection Assessment

moDC were adhered overnight to poly-L-lysine-coated eight-well glass chamber slides (SPL Life Sciences Co., Pocheon-si, Republic of Korea). On the next day, cells were pre-treated with Akt and Erk inhibitors and then infected with *L. mexicana* as described for Western blot and flow cytometry experiments. Afterwards, cells were fixed with methanol and stained with Giemsa stain (Sigma-Aldrich; Merck KGaA, Darmstadt, Germany) to assess infection with *L. mexicana*. Slides were examined with an Olympus CKX41 microscope equipped with an Olympus EC50 camera (Olympus, Tokyo, Japan). To determine parasite loads in moDC, 200 cells from random fields per condition were counted using the ImageJ software (National Institutes of Health, Bethesda, MD, USA), and the average number of intracellular amastigotes was calculated using the following formula: (# parasites/# infected cells) X (# infected cells/# total cells) × 100. Parasite loads were expressed as the average number of intracellular amastigotes × 100 infected cells. Also, percentages of infected cells per condition were determined. 

### 2.5. Western Blot for Akt and ERK 1/2

Infected and stimulated moDC, as previously described, were lysed to analyze the phosphorylation of the kinases Akt and ERK. Briefly, moDC with the different treatments were washed with D-PBS at 300× *g* for 10 min at 4 °C. Afterwards, they were incubated with 30 μl of lysis buffer (25 mM Tris-HCL, pH 7.6, 150 mM NaCl, 1% NP-40, 1% sodium deoxycholate, and 0.1% SDS) and supplemented with EDTA-free phosphatase inhibitor cocktail (Roche Diagnostics GmbH, Mannheim, Germany) for 15 min. Then, samples were centrifuged at 13,000× *g* for 10 min at 4 °C, and the supernatant (total extract) was recovered and kept at −70 °C. Protein concentration was determined using a bicinchoninic acid protein assay kit (Novagen; Merck KGaA, Darmstadt, Germany). Protein profiles were resolved through 12% SDS-PAGE gels. Next, 30 μg of protein was loaded for each condition, and the electrophoresis was carried out at 120 V for 90 min. Afterwards, proteins resolved in the acrylamide gel were electro-transferred to Immobilon-P (Millipore; Merck KGaA, Darmstadt, Germany) membranes in a semi-dry electro-transference chamber (Bio-Rad Laboratories, Hercules, CA, USA) at 20 V for 45 min. Following the transference, membranes were incubated in 5% nonfat dried milk (Bio-Rad Laboratories, Hercules, CA, USA) and dissolved in TBST (Tris-HCl 10 mM, NaCl 150 mM, 0.05% Tween 20, pH 7.4) for 30 min. Then, membranes were washed 3×, 5 min each, with TBST and incubated overnight at 4 °C with the following primary antibodies: 1:1000 monoclonal anti-p-Akt (sc-293125), 1:1000 monoclonal antibody anti-p-ERK (E-4), or 1:10,000 anti-actin (Santa Cruz Biotechnologies, Santa Cruz, CA, USA) diluted in TBST containing 10% nonfat dried milk. The membranes were later washed with TBST (three times for 5 min each) and incubated for 60 min at room temperature with either 1:5000 horseradish peroxidase (HRP)-conjugated horse anti-mouse IgG or 1:10,000 HRP conjugated goat anti-rabbit IgG secondary antibodies (Cell Signaling Technology, Inc., Danvers, MA, USA) diluted in TBST containing 5% nonfat dried milk. After incubation with secondary antibodies, membranes were washed with TBST (three times for 10 min each) and developed with a chemiluminescent substrate for HRP (Millipore; Merck KGaA Darmstadt, Germany). Chemiluminescence was evaluated and photo-documented with a ChemiDoc MP (Bio-Rad Laboratories, Hercules, CA, USA). Densitometric analyses of bands were performed using the Image Lab 6.1 (Bio-Rad Laboratories, Hercules, CA, USA).

### 2.6. Cytometry Analysis 

moDC were infected and stimulated as previously described and were then used to analyze the presence of HLA-DR and the costimulatory molecule CD86 and stained for flow cytometry. For this purpose, moDC were recovered from the 24-well plates and washed three times with D-PBS at 300× *g* for 10 min at 4 °C. Pellets were suspended in a blocking buffer (D-PBS, 2 mM EDTA, and 0.5% BSA) and incubated for 15 min at 4 °C. Subsequently, antibodies anti-HLA-DR and anti-CD86, both coupled to FITC, were added in a 1:50 dilution and incubated for 20 min at 4 °C while protected from light. moDC were washed one last time with D-PBS at 300× *g* for 10 min at 4 °C. When cells were not read the same day, they were fixed with 4% paraformaldehyde for 20 min at 4 °C in the dark. Cells were analyzed in a flow cytometer FACSCalibur (BD Biosciences, Franklin Lakes, NJ, USA), and the data were analyzed with FlowJo™ software v10.0.7 (BD Biosciences, Franklin Lakes, NJ, USA).

### 2.7. RT-PCR for IL-12p40

moDC were pre-treated for 2 h with Akt and Erk inhibitors and then infected with *L. mexicana* parasites for 4 h. Stimulation of the cells with LPS (100 ng/mL) for 4 h in the presence or absence of parasites was used as control to induce IL-12 p40 expression. Total RNA was isolated from cells after lysis with TRI-reagent (Sigma-Aldrich; Merck KGaA, Darmstadt, Germany) according to the manufacturer’s instructions. One microgram of total RNA from each sample was retrotranscribed using oligo dT primers and SuperScript™ III Reverse Transcriptase (Gibco; Thermo Fisher Scientific, Inc., Waltham, MA, USA) following the manufacturer’s instructions. One microgram of cDNA obtained from each sample was assayed for PCR amplification of a 674 bp sequence of human IL-12 p40 gene and a 138 bp sequence of human GAPDH gene (constitutively expressed) using Phusion High-Fidelity DNA Polymerase (New England Biolabs, Inc., Ipswich, MA, USA) according to standard techniques. For PCR amplifications, the following primers were used: IL-12 p40 forward: 5′-TTT TCT GGC ATC TCC CCT CGT G-3′ and reverse: 5′-TGG GTG GGT CAG GTT TGA TGA TG-3′; GAPDH forward: 5′-GCA CCG TCA AGG CTG AGA AC-3′ and reverse: 5′-TGG TGA AGA CGC CAG TGG A-3′. Amplification products were assessed by electrophoresis on 2.0% agarose gels stained with ethidium bromide, and densitometric analysis of the bands was performed using ImageJ software (National Institutes of Health, Bethesda, MD, USA). 

### 2.8. ELISA for IL-12 

The supernatants from infected and stimulated moDC were collected and stored at –70 °C to later determine IL-12 p40 concentration. P40, without interference by IL-12 p70 protein, was determined with the capture antibody C8.3 for human IL-12p40, paired with the biotinylated C8.6 antibody as the detecting antibody, with recombinant human IL-12 p40 monomer as the standard BD (Franklin Lakes, New Jersey, USA). P70 was determined using a commercial kit (Human IL-12 p70 DuoSet^®^ ELISA Kit (R&D Systems, Inc., Minneapolis, MN, USA) and following the manufacturer’s instructions. 

### 2.9. Data Analysis 

Data are reported as the means ± standard errors of the means (SEMs) and were analyzed using GraphPad Prism 6.0 software (GraphPad Software, Inc., La Jolla, CA, USA). Statistical differences between groups were evaluated using one-way analyses of variance (ANOVAs) followed by Tukey for multi-pair comparisons. Differences between groups were considered significant when the *p*-value was <0.05. 

## 3. Results

### 3.1. L. mexicana Metacyclic Promastigotes Infect moDC after 24 h of Incubation with the Cells, Which Is Prevented with the Akt Inhibitor

**Figure 1:**
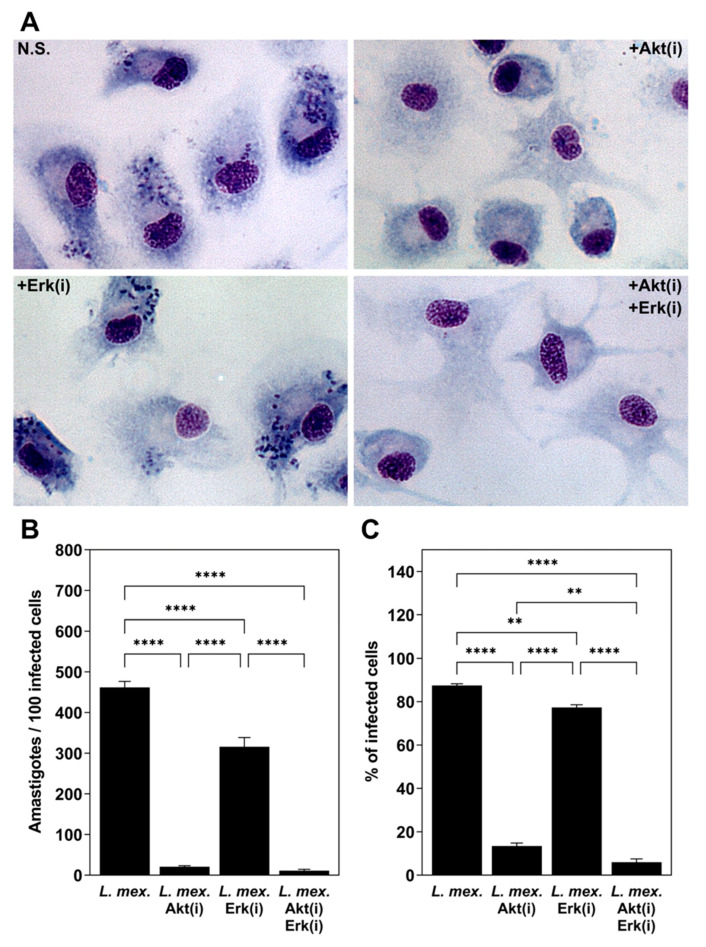
moDC were infected with *L. mexicana* metacyclic promastigotes, which was prevented when Akt was inhibited. moDC were adhered overnight to poly-L-lysine-coated eight-well glass chamber slides. On the next day, cells were pre-treated with Akt and Erk inhibitors and then infected with *L. mexicana* as described previously. Afterwards, cells were fixed with methanol and stained with Giemsa stain (**A**), photomicrographs at 400×. Parasite loads are expressed as the average number of intracellular amastigotes per 100 infected cells (**B**). Also, percentages of infected cells per condition were determined (**C**). Data are expressed as the mean ± SEM of 4 independent experiments. (** *p* ≤ 0.001; **** *p* < 0.00001).

It has been widely demonstrated that DC are infected with *Leishmania* parasites. Our group has shown the infection of moDC with *L. mexicana* promastigotes and amastigotes [49]. In the present work, we aimed to show the infection of moDC with *L. mexicana* metacyclic promastigotes after 24 h under the conditions of Akt and ERK inhibition. As shown in Figure 1A, unstimulated moDC were infected with *L. mexicana* metacyclic promastigotes, as demonstrated by the presence of several amastigotes in the cells. The infection of moDC with *L. mexicana* was not affected when cells were stimulated with an ERK inhibitor. Contrarily, the incubation of the cells with an Akt inhibitor prevented the infection of the cells with *L. Mexicana* (Figure 1B,C).

### 3.2. L. mexicana Induces Akt Phosphorylation, While the Akt Inhibitor Inhibits It

**Figure 2:**
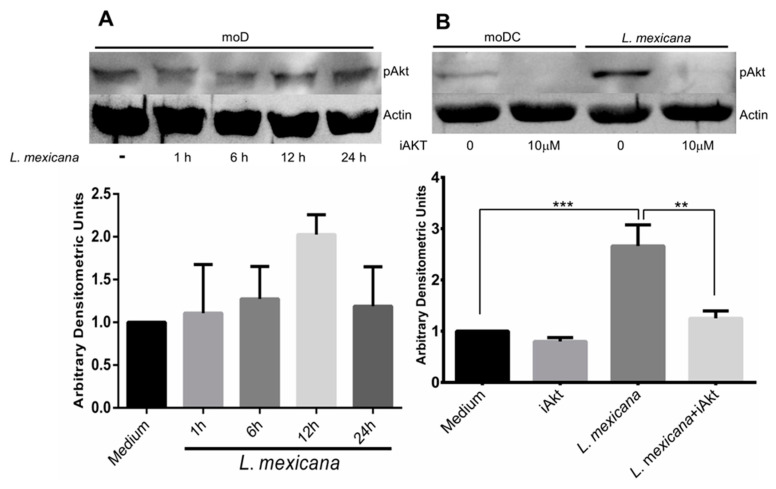
The infection of moDC with *L*. *mexicana* metacyclic promastigotes induced Akt phosphorylation, while a specific inhibitor for Akt (iAkt) prevented it. moDC were incubated in medium alone or with a specific inhibitor for Akt (iAkt) for 2 h and infected with *L. mexicana* metacyclic promastigotes. Cells were lysed, and the total extract was resolved in 12% SDS-PAGE and electro-transferred, and the membranes were incubated with anti-phospho-Akt and anti-actin. (**A**) Akt phosphorylation kinetics during 24 h. Representative blot of three independent experiments (**B**). The induced Akt phosphorylation in infected moDC was prevented with the Akt inhibitor. Data are expressed as the mean ± SEM of four independent experiments. (** *p* ≤ 0.001; *** *p* < 0.00001).

Our group previously showed, in the context of apoptosis inhibition, that the infection of moDC with *L. mexicana* amastigotes induces Akt phosphorylation [41]. In the present work, we decided to find out if Akt phosphorylation is maintained for long periods during the infection of moDC with *L. mexicana* metacyclic promastigotes and how this phosphorylation occurs over time. Our results show that metacyclic promastigotes slightly induced Akt phosphorylation starting at 1 h and maintained it until 24 h with a peak at 12 h (Figure 2A), although differences between uninfected and infected cells were not statistically significant. The incubation of moDC with the Akt inhibitor VIII drastically diminished the observed Akt phosphorylation, even though cells were infected with *L. mexicana* metacyclic promastigotes (Figure 2B).

### 3.3. L. mexicana Induces ERK Phosphorylation, While Akt Inhibitor Prevents It

**Figure 3:**
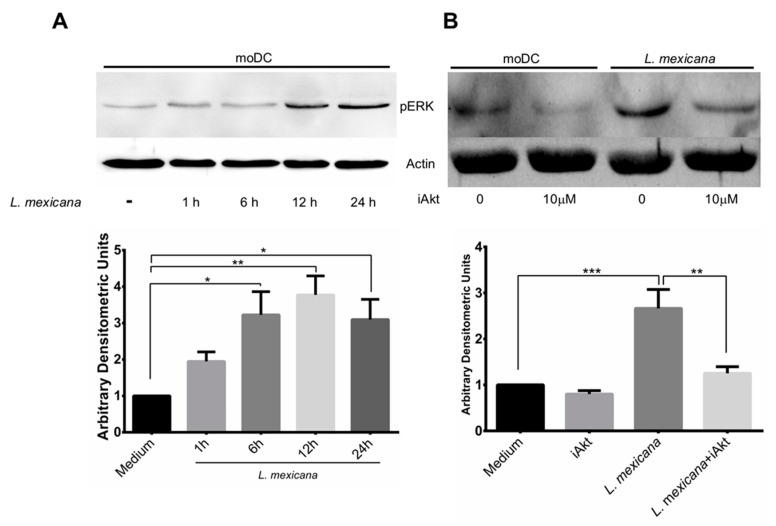
The infection of moDC with *L*. *mexicana* metacyclic promastigotes induced the phosphorylation of ERK, which diminished when Akt was inhibited. moDC were incubated in medium alone or with a specific inhibitor for Akt (iAkt) for 2 h and infected with *L. mexicana* metacyclic promastigotes. Cells were lysed, and the total extract was resolved in 12% SDS-PAGE and electro-transferred, and the membranes were incubated with the antibodies anti-phospho-ERK and anti-actin. (**A**) ERK phosphorylation kinetics during 24 h, with a peak between 12–24 h. Representative blot of five independent experiments. (**B**) The phosphorylation of ERK induced by the infection of moDC with *L. mexicana* was prevented when Akt was inhibited with a specific inhibitor. Data are expressed as the mean ± SEM of five independent experiments. (* *p* < 0.05; ** *p* ≤ 0.01; *** *p* < 0.0001).

DC maturation is a highly regulated process both at the genetic level as well as at the signaling pathways. It has been observed that MAPK’s participation is very important in this process [19]. Considering the latter, for this particular study, we decided to analyze if the kinase ERK is modulated during the infection of moDC with *L. mexicana* metacyclic promastigotes and if this kinase is regulated by Akt. Our results show that the infection of moDC with *L. mexicana* induced the sustained phosphorylation of ERK, which as detectable starting from the first hour of infection until 24 h post infection, with a peak at 12 h (Figure 3A). Additionally, we decided to find out if the pharmacological inhibition of Akt with the inhibitor VIII also inhibits ERK phosphorylation. This was performed with the objective to determine if the inhibition of Akt affected the MAPK signaling pathway, in particular ERK. Our results show that the specific inhibition of Akt significantly diminished ERK phosphorylation, at least until 18 h of infection, which was the infection time that we evaluated (Figure 3B). This suggests that *L. mexicana* utilizes the Akt signaling pathway to modulate other pathways such as MAPK. 

### 3.4. Akt Inhibitor Reestablishes CD86 Expression in Infected moDC but Does Not Affect MHCII 

**Figure 4:**
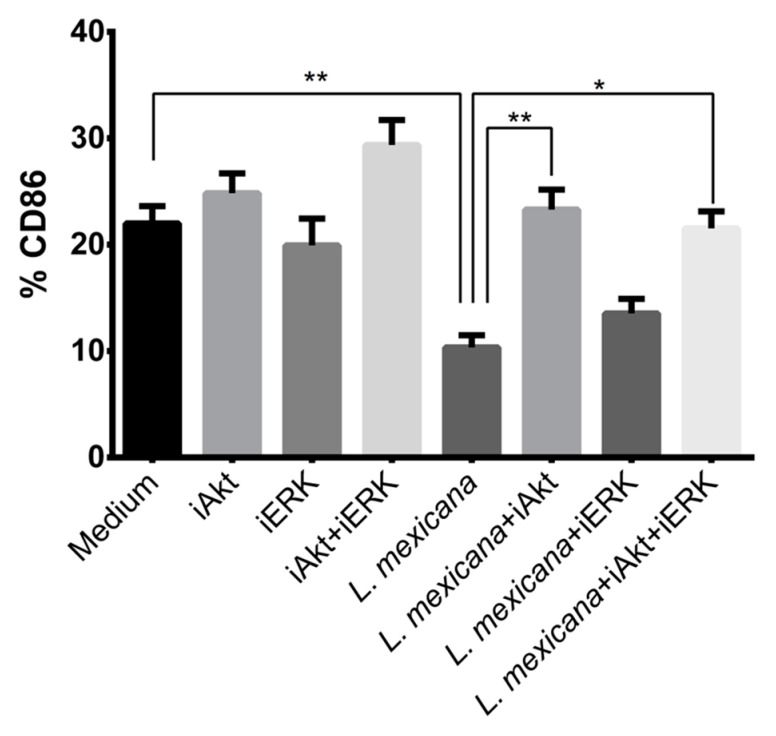
The infection of moDC with *L*. *mexicana* metacyclic promastigotes diminished the expression of the costimulatory molecule CD86, which was reestablished when Akt was inhibited. moDC were treated with specific inhibitors for ERK and Akt to determine the role of both kinases in the expression of the costimulatory molecule CD86 as a feature of moDC maturation. Cells were then infected with *L. mexicana* metacyclic promastigotes for 24 h and stained with FITC-anti-CD86 to analyze the expression of this molecule by flow cytometry. Data are expressed as media ± SEM of three independent experiments. (* *p* < 0.05; ** *p* ≤ 0.01).

**Figure 5:**
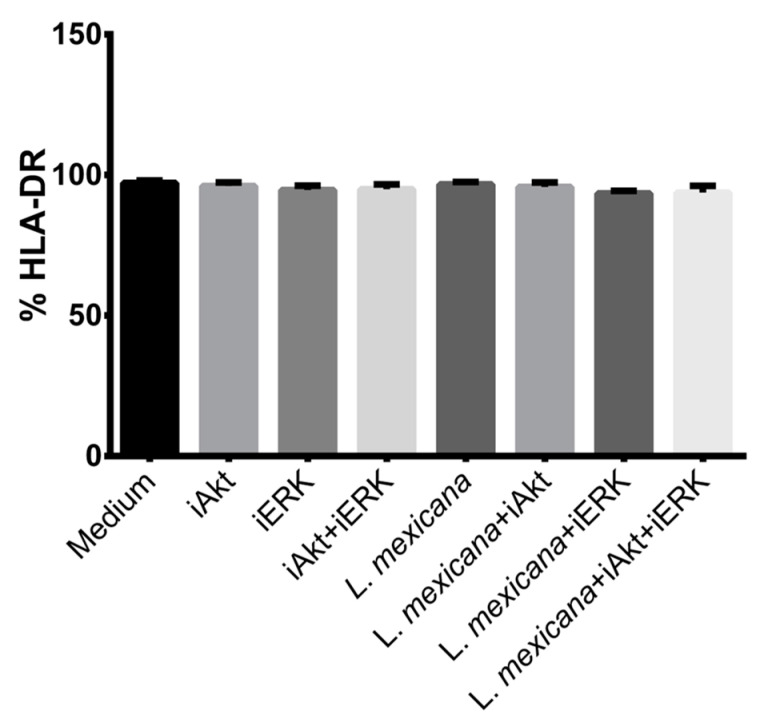
The infection of moDC with *L*. *mexicana* metacyclic promastigotes and the inhibition of Akt or ERK did not affect the presence of HLA-DR. moDC were treated with specific inhibitors for ERK and Akt to determine the role of both kinases in the expression of HLA-DR as a feature of moDC maturation. Cells were then infected with *L. mexicana* metacyclic promastigotes for 24 h and stained with FITC-anti-HLA-DR to analyze the expression of this molecule by flow cytometry. Data are expressed as media ± SEM of three independent experiments.

During DC maturation, the presence of the costimulatory molecules CD40, CD80, CD83, and CD86 is positively regulated as well as MHCII. Interestingly, it was observed that the infection of murine DC with *L. mexicana* does not increase the expression of costimulatory molecules [38,39]. Due to this finding, we performed analysis to determine whether the infection of moDC with *L. mexicana* metacyclic promastigotes regulates two important features of DC maturation: CD86 and MHCII expression. We also performed analysis to determine if, in this regulation, the kinases ERK and Akt participate. Our results revealed that the infection of moDC with *L. mexicana* significantly diminishes CD86 presence, which coincides with previous reports [37] and suggests that *L. mexicana* is a poor inducer of DC maturation. However, when Akt was pharmacologically inhibited, CD86 presence in moDC was reestablished (Figure 4 and Figure S1). This result was not observed when ERK was inhibited, since we did not observe a level of CD86 expression similar to infected moDC. Interestingly, the presence of HLA-DR in moDC is independent to the infection with *L. mexicana* and the inhibition of Akt and ERK (Figure 5 and Figure S2), which does not coincide with previous results [37]. 

### 3.5. Akt Inhibitor Reestablishes IL-12 p40 Secretion, but Not Transcription, in Infected moDC

**Figure 6:**
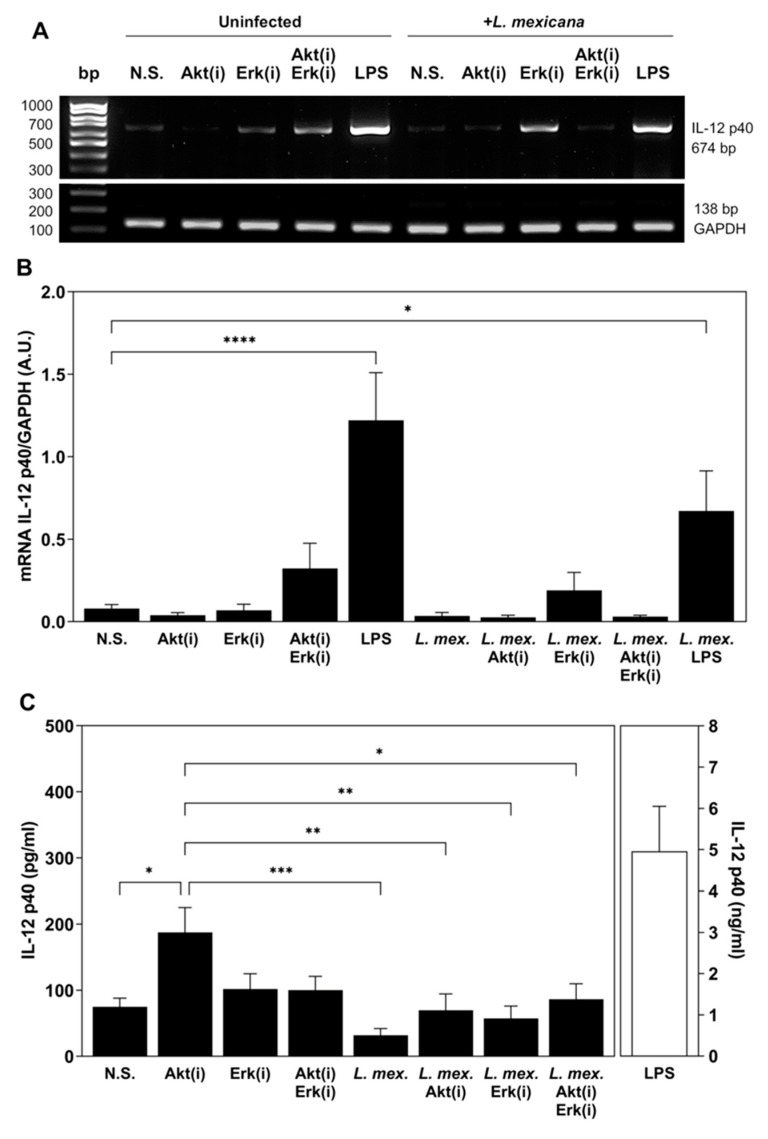
The infection of moDC with *L*. *mexicana* metacyclic promastigotes diminished IL-12 p40 Scheme 12. by moDC. Afterwards, they were infected with *L. mexicana* metacyclic promastigotes, total RNA was isolated from cells and retrotranscribed, and the cDNA obtained from each sample was assayed for PCR amplification of a 674 bp sequence of human IL-12 p40 gene and a 138 bp sequence of human GAPDH gene (**A**). Densitometric values obtained from three independent experiments are indicated (**B**). Also, culture supernatants were recovered for IL-12 p40 determination by ELISA (**C**). LPS (100 ng/mL) was used as a positive control for the production of IL-12p40. Data are expressed as media ± SEM of three independent experiments. (* *p* < 0.05; ** *p* ≤ 0.01; *** *p* < 0.0001; **** *p* < 0.00001).

Another important feature of DC maturation is the production of IL-12. IL-12 is a heterodimeric pro-inflammatory cytokine composed of the subunits p40 and p35 that together constitute the bioactive form p70. It induces the production of IFN-γ, favors differentiation of Th1 cells, and forms a link between innate resistance and adaptive immunity. DC produce IL-12 in response to pathogens during infection [50]. moDC were treated and infected, as previously described. Total RNA was isolated from cells retrotranscribed and assayed for PCR amplification of human IL-12 p40 gene. Amplification products were assessed by electrophoresis on 2.0% agarose gels stained with ethidium bromide. As shown in Figure 6A,B, unstimulated moDC did not express IL-12p40. The only condition, besides the positive control with LPS, that increased the expression of IL-12 p40 was the combination of Akt and ERK inhibitors. No infection of moDC with *L. mexicana* metacyclic promastigotes induced the expression of IL-12p40. To determine the effect of the infection of moDC with *L. mexicana* and Akt/ERK inhibition at the level of protein secretion, the supernatants of cell cultures were recovered, and IL-12 p40 was determined by ELISA sandwich. The results obtained in the ELISA assay did not coincide with the RT-PCR. Unstimulated moDC secreted a small amount of IL-12 p40, which significantly increased when cells were treated with the Akt inhibitor. The infection of moDC with *L. mexicana* diminished the production of IL-12p40 observed in unstimulated cells, and this production also increased when cells were stimulated with the Akt inhibitor and infected with *L. mexicana* (Figure 6C). 

## 4. Discussion

Dendritic cells (DC) represent a crucial cell population in *Leishmania* infection, and an effective immune response against it depends on the successful activation and maturation of DC. 

For years, it was thought that macrophages were the only cells to harbor *Leishmania* until Moll et al. [51] described that murine Langerhans cells (LCs) could ingest *L. major* and migrate to lymph nodes. Since that first description, abundant literature has demonstrated the pivotal role of DC in leishmaniasis primarily because of their function in shaping the immune response. DC are considered the most powerful antigen-presenting cells due to their capacity to prime naïve T cells [6,7]. DC traverse through a maturation process initiated primarily with the recognition of PAMPs or DAMPs that induces the activation of diverse programs with the participation of signaling pathways such as MAPK [19]. During the process of maturation, DC upregulate the expression of MHCII and costimulatory molecules and produce IL-12, which polarizes the immune response to a Th1 phenotype that is detrimental for the parasite. Thus, *Leishmania* has developed several schemes to prevent the development of a Th1 response. Numerous in vitro and in vivo studies demonstrate that successful *Leishmania* infections involve the impairment of DC functions [38,52,53,54]. Several *Leishmania* species have been shown to downregulate IL-12 secretion from macrophages and DC. Particularly, *L. mexicana* inhibits IL-12 in infected macrophages and DC [37]. Interestingly, DC, but not macrophages, recover the capacity to produce IL-12 when exposed to a stimulus such as LPS/IFN-γ [55,56,57]. Another strategy displayed by *Leishmania* to affect host cell functions is the inactivation of microbicidal mechanisms such as NO and ROS production and interference with signaling pathways in infected macrophages [35,36,58]. Additionally, *Leishmania* can also delay DC maturation to impede their transit through these powerful antigen-presenting cells by inhibiting MHCII, CD86, and CD54 expression [37]. It has been shown that the infection of CBA mice bone marrow-derived DC with *L. mexicana* amastigotes and promastigotes inhibits DC maturation. There is no increase in the presence of surface markers such as CD54, CD86, and MHC-II, and the production of IL-12 is inhibited. Interestingly, the infection of DC does not eliminate its maturation capacity since, if stimulated with LPS/IFN-γ, they manage to mature [37]. Also, it has been observed that the infection with *L*. *mexicana* promastigotes of immortalized DC derived from resistant C57BL/6 mice also inhibited DC maturation. This has been associated with a rapid activation of tyrosine phosphatases that could be directly related with MAPK along with the inhibition of transcription factors such as AP-1 and NF-κB [39]. Recently, it was demonstrated that the infection of DC with *L. amazonensis* modifies some transcription factors. Specifically, the infection activates the alternative NF-kB pathway and inhibits the canonical NF-κB pathway as well as DC maturation and inflammasome activation [59]. 

We previously showed that the infection of moDC with *L. mexicana* amastigotes or promastigotes intervenes with the signaling pathways of p38 and JNK MAPK and PI3K/Akt. In the case of p-38 and JNK, infection with both phases of the parasite diminishes the phosphorylation of both kinases, which contributes to the inhibition of moDC apoptosis exerted by *L. mexicana*. Contrarily, amastigotes induce the activation of PI3K/Akt to prolong moDC survival and perpetuate infection [40,41]. On the other hand, it was shown that the infection of murine macrophages with *L*. *amazonensis* induces a sustained activation of Akt, and the pharmacological inhibition of this pathway restores IL-12 production [42]. In the present work, we decided to study if *L. mexicana* metacyclic promastigotes regulate moDC maturation through Akt and ERK signaling pathways. Our first approach was to obtain monocyte-derived dendritic cells (moDC) and infect them with *L. mexicana* metacyclic promastigotes. We previously demonstrated that *L. mexicana* amastigotes upregulated Akt phosphorylation as a strategy to prevent moDC apoptosis [41]. Both *Leishmania* developmental stages present important differences, and it has been shown that they are involved in the role that DC play during *Leishmania* infection [46,47,48]. Thus, we considered that it was relevant to demonstrate the effect of metacyclic promastigotes, the most infective stage, in the phosphorylation of Akt and its role in DC maturation. Before this, we analyzed the effect of an Akt inhibitor and an ERK inhibitor in the infection of moDC with *L. mexicana*. We previously showed that *L. mexicana* promastigotes and amastigotes infect moDC [49]. We performed infection assays in poly-L-lysine-coated chambers and demonstrated by Giemsa staining that moDC are infected with *L. mexicana* metacyclic promastigotes. Interestingly, the infection of moDC with the parasites was prevented when cells were stimulated with an Akt inhibitor but not with an ERK inhibitor. It is known that PI3K is a key regulator of phagocytosis, and Akt is one of the main signal transducers activated by PIP_3_ produced during the activation of PI3K [60,61]. It was also shown that inhibitors of PI3K activation interfered with dengue virus infection [62]. Also, the inhibition of Akt with inhibitor VIII diminished, in a dose-dependent manner, the infection of THP-1 cells with pneumococci [63]. On the other hand, *Trypanosoma* extracellular amastigotes activate Akt and ERK during HeLa cells invasion. The inhibition of both kinases prevented the infection process [64]. These results coincide with our findings, although the inhibition of ERK did not prevent the infection of moDC with *L. mexicana*. Once we showed that Akt participates in the infection of moDC with *L. mexicana* metacyclic promastigotes, we pursued analysis to determine the effect of the parasite in the phosphorylation of Akt. Stimulated and infected cells were lysed, and protein extracts were tested for Akt phosphorylation. We showed that *L. mexicana* metacyclic promastigotes induce Akt phosphorylation, while the Akt inhibitor inhibits it. The activation of Akt coincides with our previous finding with *L. mexicana* amastigote infection of DC [41] and what has been demonstrated in macrophages infected with *L. amazonensis* [42]. Then, we aimed to investigate the effect of the infection of moDC with *L. mexicana* metacyclic promastigotes in the activation of ERK. We previously showed that the infection of moDC with *L. mexicana* induces the phosphorylation of p38 and JNK as a mechanism to inhibit host apoptosis [28] and also that *L. mexicana* LPG activates ERK and p38 [65]. 

We showed that the infection of moDC with *L. mexicana* metacyclic promastigotes phosphorylates ERK, and the presence of a specific Akt inhibitor diminishes ERK phosphorylation in infected cells, which presupposes a coordinated action between Akt and ERK during *L. mexicana* infection of moDC. The MAPK and Akt signaling pathways are involved in different pathogen-triggered immune responses [66]. MAPK signaling pathway regulates cell growth and differentiation and controls cellular responses to cytokines and stress [67]. The PI3K/Akt signaling pathway mainly participates in cell survival [68]. Although these signaling pathways transit in parallel, several interconnections have been described [33]. It was shown that Akt can have a positive transcriptional control of ERK. Akt can downregulate p38 and glycogen synthase kinase 3 (GSK3), which at the same time diminishes MAPK phosphatases (MKP) expression and prolongs ERK phosphorylation [33]. We then aimed to investigate if the activation of both Akt and ERK in *L. mexicana*-infected moDC had an effect on cell maturation. Human DC are identified by their high expression of major histocompatibility complex (MHC) class II molecules, and during the process of maturation, they overexpress costimulatory molecules such as CD80 and CD86. We analyzed the expression of CD86 and MHCII in moDC treated with or without Akt or ERK inhibitors. We showed that the infection of moDC with *L. mexicana* downregulated the expression of CD86 but not of MHCII. Notably, when cells were treated with the Akt-specific inhibitor, the expression of CD86 was restored to levels similar to basal ones. A similar result was shown in the infection of BMDC with *L. infantum*, where the inhibition of PI3K/Akt by wortmannin before BMDC infection caused a slight increase in the transcription of costimulatory molecules CD40 and CD86 [69]. Our results suggest *L. mexicana* targets Akt to downregulate DC maturation, and when Akt is inhibited, some features of DC maturation such as CD86 expression are restored. 

As already mentioned, the production of IL-12 is an important feature of DC maturation. IL-12 induces the production of IFN-γ, favors the differentiation of Th1 cells, and forms a link between innate resistance and adaptive immunity [50]. Thus, IL-12 production is detrimental for the parasite, and it has developed different strategies to dampen it. However, there are marked differences in the modulation of DC IL-12 production by distinct *Leishmania* species. DC infected with *L. major* or *L. donovani* produce high levels of IL-12, while infection of DC with *L. amazonensis* induces low amounts of this cytokine. We tested the transcription and production of IL-12 p40 in moDC treated with the inhibitors for ERK and Akt and infected with *L. mexicana* metacyclic promastigotes. We observed that the expression of IL-12 p40 at the transcription level did not coincide with the protein levels determined by ELISA. By RT-PCR, the only increase in IL-12 p40 besides the positive controls with LPS was when uninfected cells were treated with both inhibitors. Contrarily, when the levels of IL-12 p40 were determined by ELISA, we found that the secretion of IL-12 p40 was low, although in our experience *L. mexicana* is a not a good inducer of IL-12 production, but very interestingly, the inhibition of Akt was the condition that caused the major IL-12 p40 production. This coincides with the result of CD86 expression and supports the idea that *L. mexicana* targets Akt to affect DC maturation. The difference in the results between IL-12 p40 expression and secretion suggests that post-transcriptional regulation is occurring. In addition to the role of Akt in the expression of CD86 and IL-12 in DC that we observed, it was also shown that these two molecules, among others, can also be affected by the microenvironment, for example, oxygen concentration. It was shown that under hypoxia, there is a reduction in CD80 and CD86 expression and an increase in the production of IL-12p70, which has an impact in the control of the infection of DC with *L. amazonensis* [70]. 

With the results of the present work and the previously published information, it is possible to establish that the regulation of the kinase Akt is crucial during the infection of moDC with *L. mexicana*. In Mexico, this species is the principal causal agent of cutaneous leishmaniasis, which in patients can manifest in two diametrically different forms: localized cutaneous leishmaniasis (LCL), where an ulcer forms at the sites of parasite inoculation, and diffuse cutaneous leishmaniasis (DCL), characterized by multiple disfiguring nodules due to the spreading of parasites throughout the skin [71,72]. The factors underneath the development of such different clinical forms caused by *L. mexicana* have not been fully deciphered. There is an association of a Th1 immune response against *L. mexicana* infection and low macrophage parasite loads in patients with LCL. Contrarily, patients with DCL show a polarized Th2 response and high macrophage parasite loads [73,74,75]. Due to the fact that it is the same species that causes these opposed clinical manifestations, it is possible that something intrinsic to the parasite may influence the fate of the disease. We previously showed that two different *L. mexicana* isolates, one obtained from a patient with LCL and one from a patient with DCL, behave in a distinct manner both in culture as well as in the regulation of Arg-1 and NOS2 [76]. We also tested these isolates in their capacity to inhibit apoptosis and also observed differences: the most virulent isolate was capable of inducing more arginase expression [76] and apoptosis inhibition (unpublished results). These results permit us to speculate that the manipulation of Akt by *L. mexicana* could be another virulence trait that can contribute to the development of LCL or DCL. It will be very interesting to analyze the effect of different *L. mexicana* isolates in the maturation of moDC and the regulation of Akt. 

Other factors were recently shown to participate in the maturation of DC and concomitant parasite elimination, such as the interaction through DC-SIGN of neutrophils with *L. amazonensis*-infected DC [77]. 

## Data Availability

The original contributions presented in the study are included in the article/supplementary material, further inquiries can be directed to the corresponding author.

## Supplementary Materials

The following supporting information can be downloaded at: https://www.mdpi.com/article/10.3390/tropicalmed9050118/s1, Figure S1. The presence of CD86 in moDC infected with *L. mexicana* metacyclic promastigotes diminished as compared to uninfected moDC. The specific inhibition of Akt increased the expression of CD86, while the specific inhibition of ERK did not increase the expression of CD86. Representative cytometry histograms of three independent experiments. The light-gray profile represents unstained moDC; Figure S2: The antigen-presenting molecule HLA-DR is constantly expressed in moDC independently of the infection with *L. mexicana* metacyclic promastigotes or the specific inhibition of Akt or ERK. Representative cytometry histograms of three independent experiments. The light-gray profile represents unstained moDC.
