# Supplementary material for: Human Dendritic Cell Maturation Is Modulated by Leishmania mexicana through Akt Signaling Pathway
Source: Trop Med Infect Dis. 2024 May 17;9(5):118. doi: 10.3390/tropicalmed9050118 (PMC11126033; doi:10.3390/tropicalmed9050118)
Supplement: Supplementary file 1 [file tropicalmed-09-00118-s001.zip › tropicalmed-2798334-supplementary.pdf]

Tropical Medicine and Infectious Disease

MDPI

Supplementary Figures

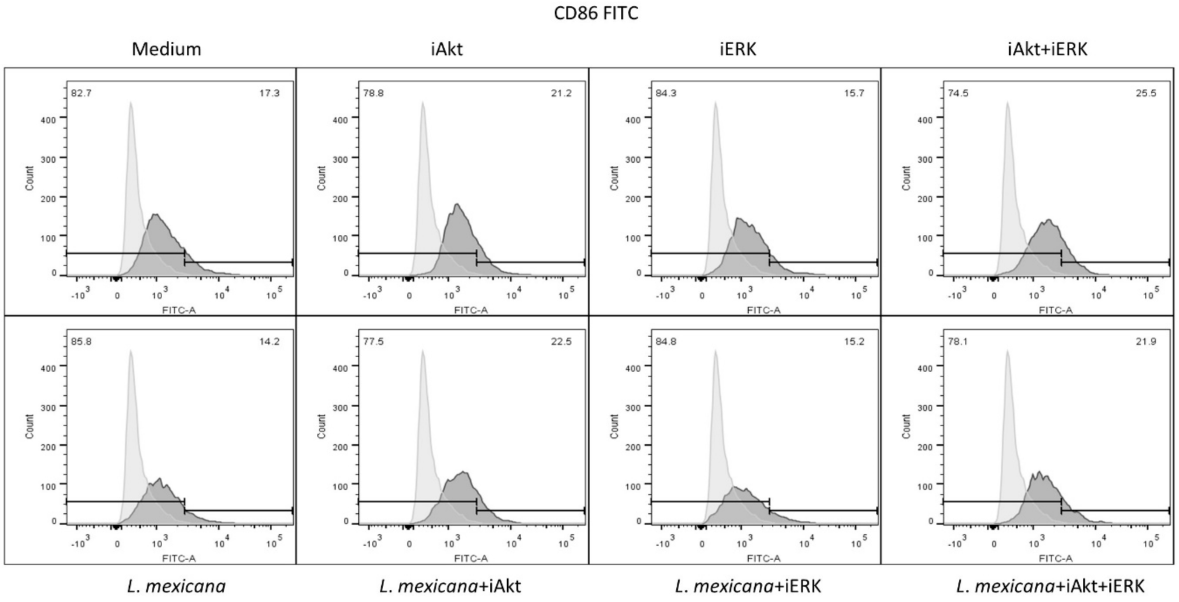

Figure S1. The presence of CD86 in moDC infected with *L. mexicana* metacyclic promastigotes diminished as compared to uninfected moDC. The specific inhibition of Akt increased the expression of CD86, while the specific inhibition of ERK did not increase the expression of CD86. Representative cytometry histograms of three independent experiments. The light-gray profile represents unstained moDC.

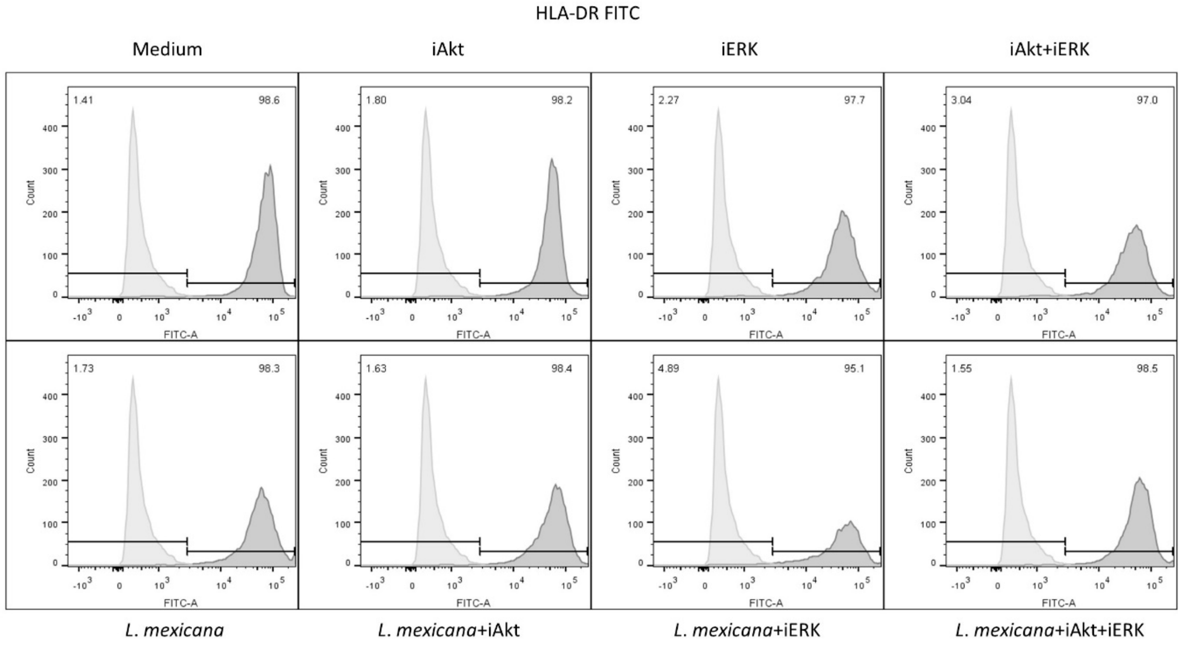

Figure S2. The antigen-presenting molecule HLA-DR is constantly expressed in moDC independently of the infection with *L. mexicana* metacyclic promastigotes or the specific inhibition of Akt or ERK. Representative cytometry histograms of three independent experiments. The light-gray profile represents unstained moDC.
